# Mapping ultra-processed foods (UPFs) in India: a formative research study

**DOI:** 10.1186/s12889-024-19624-1

**Published:** 2024-08-14

**Authors:** Suparna Ghosh-Jerath, Neha Khandpur, Gaurika Kumar, Sahiba Kohli, Meenu Singh, Inderdeep Kaur Bhamra, Fernanda H Marrocos-Leite, K Srinath Reddy

**Affiliations:** 1 The George Institute for Global Health, 308, Third Floor, Elegance Tower, Plot No. 8, Jasola District Centre, New Delhi, Delhi 110025 India; 2 Wageningen University, Wageningen, The Netherlands; 3 Public Health Foundation of India, New Delhi, India; 4 Center for Epidemiological Research in Nutrition and Health, Faculty of Public Health, University of Sao Paulo, Sao Paulo, Brazil

**Keywords:** Ultra-processed foods, Nova food classification, UPF categories, Online grocery retailer scan, Tool adaptation, Nova-UPF screener, India

## Abstract

**Background:**

Increased consumption of ultra-processed foods (UPFs) which have additives such as artificial colours, flavours and are usually high in salt, sugar, fats and specific preservatives, are associated with diet-related non-communicable diseases (NCDs). In India, there are no standard criteria for identifying UPFs using a classification system based on extent and purpose of industrial processing. Scientific literature on dietary intake of foods among Indian consumers classifies foods as unhealthy based on presence of excessive amounts of specific nutrients which makes it difficult to distinguish UPFs from other commercially available processed foods.

**Methods:**

A literature review followed by an online grocery retailer scan for food label reading was conducted to map the types of UPFs in Indian food market and scrutinize their ingredient list for the presence of ultra-processed ingredients. All UPFs identified were randomly listed and then grouped into categories, followed by saliency analysis to understand preferred UPFs by consumers. Indian UPF categories were then finalized to inform a UPF screener.

**Results:**

A lack of application of a uniform definition for UPFs in India was observed; hence descriptors such as *junk-foods*,* fast-foods*,* ready-to-eat foods*,* instant-foods*,* processed-foods*,* packaged-foods*,* high-fat-sugar-and-salt foods* were used for denoting UPFs. After initial scanning of such foods reported in literature based on standard definition of UPFs, an online grocery retailer scan of food labels for 375 brands (atleast 3 brands for each food item) confirmed 81 food items as UPFs. A range of packaged traditional recipes were also found to have UPF ingredients. Twenty three categories of UPFs were then developed and subjected to saliency analysis. Breads, chips and sugar-sweetened beverages (e.g. sodas and cold-drinks) were the most preferred UPFs while frozen ready-to-eat/cook foods (e.g. chicken nuggets and frozen kebabs) were least preferred.

**Conclusion:**

India needs to systematically apply a food classification system and define Indian food categories based on the level of industrial processing. Mapping of UPFs is the first step towards development of a quick screener that would generate UPF consumption data to inform clear policy guidelines and regulations around UPFs and address their impact on NCDs.

**Supplementary Information:**

The online version contains supplementary material available at 10.1186/s12889-024-19624-1.

## Background

Non-communicable diseases (NCDs) are one of the leading causes of premature morbidity and mortality resulting in over 7 out of 10 deaths worldwide [1]. Mortality due to NCDs has been on the rise in India, increasing from 37.9% of all deaths in 1990 to 61.8% in 2016 [2, 3]. Overweight/obesity have been identified as a contributing factor [4]. The recent national-level data shows an increase of 25% in the prevalence of overweight and obesity among Indian men and women over 14–15 years and 3% among children under five years [5, 6]. Due to their thin fat phenotype, Indian infants and children, who comprise almost one quarter of the total population, are predisposed to obesity [7, 8]. These risk factors are further amplified by changing food environments and behavioural variables such as tobacco, alcohol, drug use and low physical activity [9]. Exposure to unhealthy food environments in genetically predisposed children, along with other behavioural risk factors, increases their risk of developing obesity and diet-related non-communicable diseases (DR-NCDs) in the long term [10].

The rapidly changing food environment is characterized by diets transitioning from minimally-processed staple foods (such as pulses and whole cereals) high in vitamins, minerals and fibre to refined, processed and ultra-processed foods (UPFs) [11]. The Indian population is exposed to a wide variety of UPFs which are hyper-palatable, packaged, convenient, affordable and have a long shelf life, such as sugar-sweetened beverages, chips, biscuits and bread, and ready-to-eat/ ready-to-cook (RTC) meals [12]. The sales data of UPFs in India demonstrates an exponential increase, from USD 0.9 billion in 2006 to USD 37.9 billion in 2019 [13]. This growth indicates a notable expansion of these food products in the market, coupled with widespread advertising efforts that specifically target vulnerable populations, including children and youth [14–18]. Consumer demand for UPFs has increased due to higher disposable incomes, nuclear families, single-member households, and less availability of time for housework [19, 20]. UPFs have penetrated the rural boundaries of the country and are likely making their way into households of diverse geographic and socio-economic attributes [21, 22].

The Nova food classification system categorizes foods based on the purpose and the level of processing and includes four categories: (i) unprocessed/ minimally processed foods, (ii) processed culinary ingredients, (iii) processed foods, and (iv) ultra-processed foods [23–26]. UPFs are a category of food that undergo a series of industrial processes like extrusion and moulding, and have presence of classes of additives whose function is to make the final product palatable or more appealing, such as flavours, flavour enhancers, colours, emulsifiers, thickeners, sweeteners, etc. Although not unique to UPF, they also include additives that prolong the product duration and protect original properties or prevent proliferation of microorganisms [23–26]. In addition to this, several of these products are high in saturated fats or trans-fats, added sugars, and salt and low in dietary fibre, various micronutrients and other bioactive compounds [27–35].

Overconsumption of UPFs has been associated with higher body mass index (BMI), obesity, type-2 diabetes, hypertension, cardiovascular diseases, and certain types of cancers [36–38]. Given the diversity in UPFs, there is a need to systematically map the range of UPFs accessed by the Indian population. This is an important first step to understanding their potential role in contributing to the NCD burden in India and in developing strategies to encourage the substitution of the most frequently consumed UPFs to healthier alternatives. Identifying specific UPF categories could also help inform the development of dietary assessment instruments like food frequency questionnaires (FFQ) and screeners.

## Methods

**Fig. 1:**
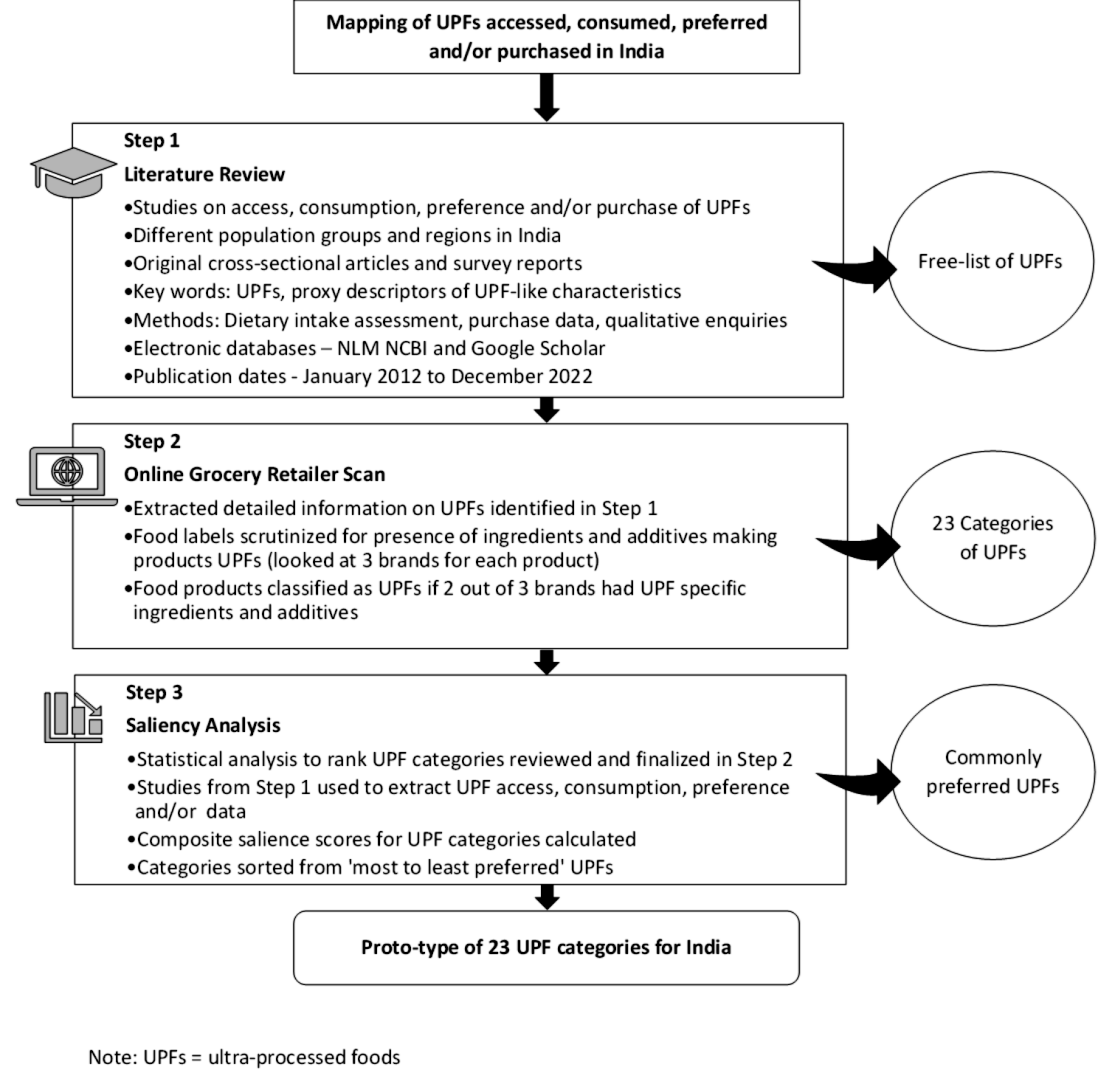
Flow chart briefly explaining the 3 steps of methodology

The present study aimed at: (i) mapping the specific categories of UPFs accessed and consumed in India, (ii) assessing the ingredient composition of these products, (iii) ranking the UPFs by consumer preference, and (iv) developing a list of categories of UPFs commonly consumed in India. For this, a secondary review of available literature complemented by an online grocery retailer scan and a saliency analysis were conducted between April 2021 and February 2022 (Fig. 1).

Step 1. The literature review was conducted to map and identify the various types of UPFs accessed, consumed, preferred and/ or purchased (as reported behaviours) in India. This review included published cross-sectional and observational research studies that used surveys, focus group discussions and interviews to elicit reported behaviours across different population groups and regions in India. International and national survey reports on UPF food intake and purchase among Indian population were also included in the review. Articles for review were identified from two electronic databases (NLM NCBI and Google Scholar). To ensure the search captured the diversity of UPFs, search terms included proxy descriptors identified in Indian policy documents [39–44], including: junk food*, fast food*, modern food*, westernized food*, ultra-processed food*, UPF*, convenience food*, ready-to-eat food*, ready-to-eat snack*, ready-to-cook food*, instant food*, frozen food*, canned food*, tinned food*, processed food*, packaged food*,* high fat*, sugar and salt food* and HFSS*. The literature search and data extraction was conducted by two authors (MS and GK).

To be eligible for the review, studies needed to: (i) include UPFs or their proxy descriptors, with examples of products, (ii) be conducted in either rural and/or urban areas of India, (iii) be published in the English language, between January 2012 and December 2022. This time frame was chosen to capture the high growth in UPFs sales during this decade [45, 46]. Review articles, and publications that did not define the food category studied or did not cite any examples of foods were excluded.

Data from eligible articles were extracted in MS-Excel to record key variables on UPFs or their proxy descriptors with examples, location of the study (national/specific state), geographical area (urban/rural), sample size, sampling method, study participants’ age (years) and dietary data collection tools such as 24 h dietary recalls, FFQ, interviews and structured questionnaires (Additional file 1). A free list of UPF foods and beverages identified from the reviewed studies, was developed.

Step 2. An online grocery retailer scan for extracting detailed information on the UPFs identified in Step 1, was also conducted. The objective was to review and scrutinize the ingredient list provided in the food labels and to confirm that the food item qualified as UPFs. For this online scan, three researchers (GK, IKB, MS) reviewed the online grocery websites of the largest grocery retailers in India - Big Basket, Grofers, and Amazon [47]. Individual foods and beverages from Step 1, were checked for their ingredient composition and the presence of additives. This activity was guided by the FAO document ‘Ultra-processed foods, diet quality, and health using the Nova classification system’ [23–26] and the expertise of the co-authors (NK and FHML). Food items were specifically scrutinized for the use of additives (flavours, flavour enhancers, colours, emulsifiers, emulsifying salts, artificial sweeteners, thickeners, foaming, anti-foaming, bulking, carbonating, gelling and glazing agents), specific ingredients such as industrially derived sugars (fructose, invert sugar, maltodextrin, dextrose, lactose, high fructose corn syrup, fruit juice concentrates), modified oils (hydrogenated fats, interesterified fats), extracted proteins (hydrolysed proteins, soy protein isolate, gluten, casein, whey protein, mechanically separated meat) [23–26, 48]. All food items were assessed for at least three different brands and if a majority of the items (2 out of 3) qualified as UPFs, the product category was confirmed as UPF. The free-list of UPFs identified from the literature and confirmed through label reading using the online grocery retailer scan were then categorized on the basis of the primary ingredient of their composition and/or functionality of the product. A 23 category UPF list was developed at the end of Step 2.

**Fig. 2:**
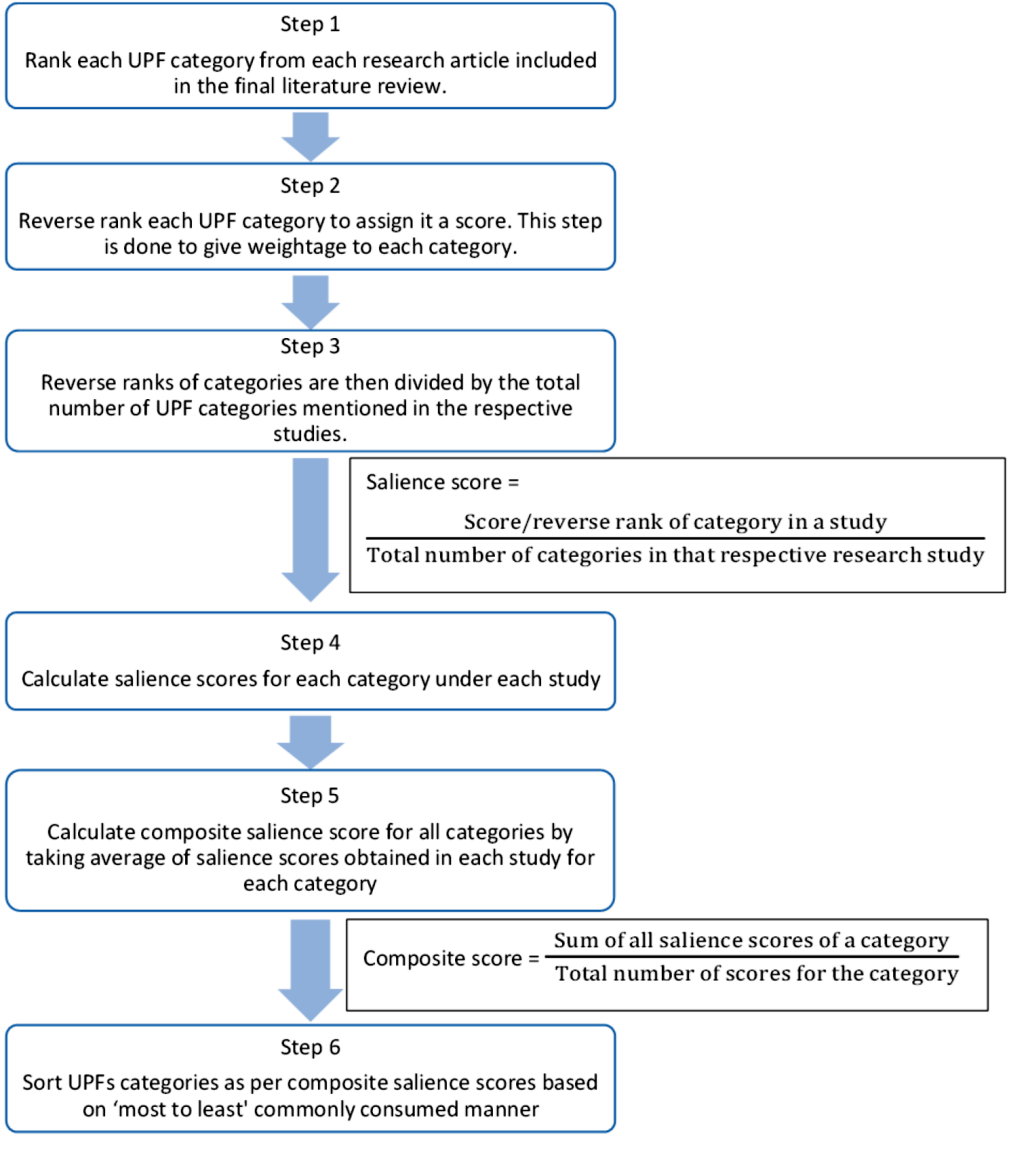
Saliency analysis method for free-listed UPF categories

Step 3. The confirmed UPF categories (23 categories), were then subjected to a saliency analysis, conducted by two authors (GK, MS). Saliency is a statistical accounting of items for rank and frequency of mention, across all respondents within a given domain. For example, the colour chosen most often from a free list of ten colours by a study population is referred to as the most salient [49]. The saliency test indicators included the commonly accessed, consumed, preferred and/or purchased (collectively referred to as ‘preferred’ in this paper to identify the common UPF categories accessed by the Indian consumers). These categories were limited to the food items that were confirmed as UPFs in Step 2. For example, if a study used “junk food” as a descriptor of UPFs and included freshly prepared savouries like *“samosa/kachori”* along with chips and soft drinks, we included data for only chips and soft drinks for the purpose of saliency analysis. The UPFs were then sorted from the most to the least preferred UPFs (Additional file 2). The steps and formulas [49] used to calculate composite salience scores for each UPF category have been illustrated in Fig. 2. The UPF categories were classified per consumer preference, to the composite salience score cut-offs, defined after dividing the distribution of the composite salience scores obtained into tertiles as follows: (i) ≥ 0.61 as frequently preferred, (ii) 0.61 − 0.51 as infrequently preferred, and (iii) < 0.51 as rarely preferred UPFs.

## Results

**Fig. 3:**
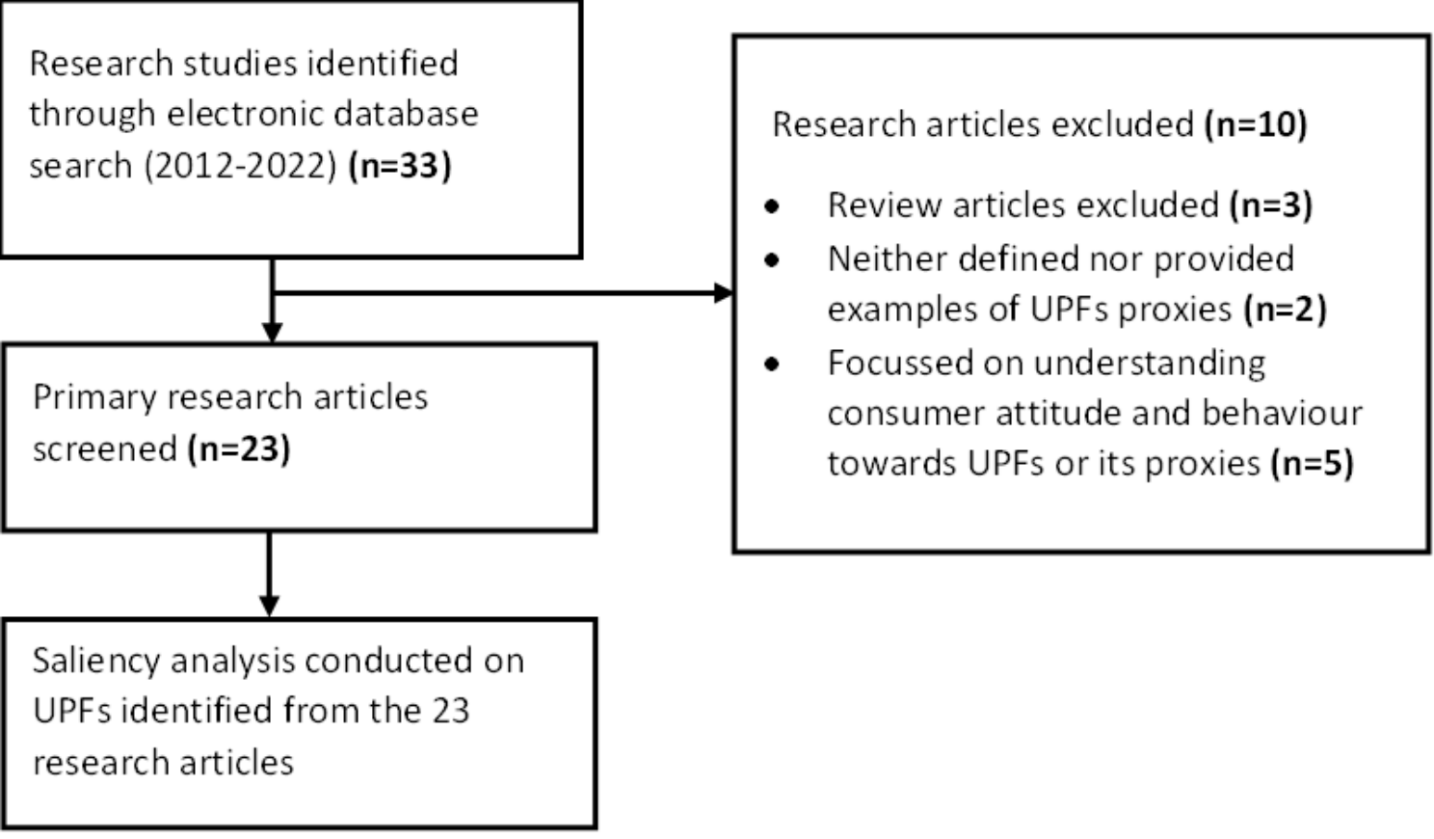
Flow diagram reporting the screening and selection of studies reporting consumption and availability of UPFs in India

The literature search and study selection process of Step 1 is illustrated in Fig. 3. A total of 23 research articles that matched the inclusion criteria were included in the final review. An overview of the extracted variables is provided in Additional File 1. These studies were conducted in both rural (5 out of 23) and urban areas (17 out of 23), in different regions of the country, among a diverse population aged between 9 and 69 years (Table 1). Table 2 provides the outcome of the literature review with proxy descriptors along with the food items listed under them. These foods were verified as UPFs and non-UPFs.

**Table 1:** Characteristics of the reviewed studies (*n* = 23)

Region	Number of studies	Geographical area				Age group (years)
		Rural	Urban	Both	Not Defined	
East	2	0	1	1	0	12–18
West	3	0	3	0	0	10–24
North	5	1	4	0	0	9–20
South	9	1	5	0	3	11–60
PAN India	4	0	1	2	1	10–69
TOTAL	23	2	14	3	4	9–69

**Table 2:** Proxy descriptors of Ultra-Processed Foods (UPFs), and categorization of food items as UPFs and non-UPFs (outcome of literature review) (*n* = 23)

Proxy descriptors	Foods and beverages identified as UPFs	Foods and beverages identified as non-UPFs	References
**Ultra-processed foods**	Chips, cake, ready-to-serve beverages, beverage concentrates, bakery products and confectionery	-	[50]
**HFSS**	*Namkeen*, packaged chips, soya or mustard sauce, processed or packaged cheese	*Papad, namkeen* (if freshly prepared), butter	[51]
**Processed foods**	**Sweet snacks**: chocolate, biscuits, cookies, jams, peanut butter, chocolate spread, *rusk*	Honey	[52]
	**Salty snacks**: crackers, potato chips, banana chips, and other salty snacks	-	
	**Soft drinks**: carbonated drinks, juices, milk-based drinks, squashes and powdered drinks	-	
	**Milk**: milk powder	Liquid milk	
	**Dairy products**: cheese	Butter, *ghee* (clarified butter)	
	**Edible oils**: *vanaspati* (partially hydrogenated vegetable oil)	Edible oils	
	**Processed wheat**: bread	*Atta* (Processed wheat), vermicelli, pasta	
	**Other processed foods**: soup, ready-to-eat (RTE) meals, ready-to-cook (RTC) mix, frozen food, breakfast cereals, noodles, ketchup, table sauces	Breakfast cereals (if not coated/extruded)	
	Noodles, *gobi manchurian*, fried rice, cola beverages, fruit beverages, *bonda, vada*, chips, puffs, cake, bread, *rusk*, bun	*Masala puri, churmuri, panipuri*, fried rice (if freshly prepared), coffee, tea, *kharasev, kodubale, chakli, bajji, bonda, vada* (if freshly prepared), puffed rice	[21]
**Packaged foods**	Ready meals, sauces, dressings and condiments, soup, sweet spreads, baby foods, dairy	Edible oils	[46]
	Confectionery, ice cream and frozen, desserts, savoury snacks, sweet biscuits, snack bars and fruit snacks, baked goods, breakfast cereals, processed meat and seafood	Plain (not coated/extruded) breakfast cereals, processed fruits and vegetables	
	-	Rice, pasta, and noodles	
**Junk foods**	Burger, pizza, pasta, instant noodles	-	[6]
	Chips, chocolates, bakery products (pastries, cream rolls, patties), soft drinks, sweetened beverages (squashes, sweet juices), ice cream	Sweets (*ladoo, jalebi, barfi*) and *samosa* (if freshly prepared)	[22]
	**South Indian**: *Idli, dosa, vada, uttapam* (RTE/RTC)	*Idli, dosa, vada, uttapam* (if freshly prepared)	[53]
	**Bakery items**: Biscuits, bread, cake, pastry, patties	-	
	**Italian food**: Pizza, pasta, macaroni	-	
	**Chinese**: *Maggi*, *manchurian*, noodles, spring roll	-	
	**Sweet dish**: Chocolate, *halwa*, ice cream, *mithai*	-	
	**Fried food**: burger, potato chips, RTE *samosa, RTE tikki*	Bread *pakora, chaat, kachori, kulcha chana*, potato chips, freshly prepared *samosa,* freshly prepared *tikki*	
	**Beverages**: carbonated drinks, juices	Tea, coffee	
	Ice-creams, sweets, chocolates, pizza, carbonated beverages	Fried and bakery foods (if freshly prepared), fruits	[54]
	Pizza, burger, puffs, pastry, cake, biscuits, French fries, noodles, doughnuts, Pepsi, Coca-Cola, Slice, 7-UP, Bovonto, Sprite	-	[55]
	Cakes, chocolates, ice-creams, chips, *Kurkure*, street fast foods, noodles	Sweets, Indian deep-fried foods, street fast foods (if freshly prepared)	[56]
**Indian fast foods**	Pizza, burger, French fries, donuts, hot dog, noodles, cold drinks, carbonated soda, health drinks	*Chat, pakora, samosa*, patties, *panipuri, puri-kachori, chole bhature*, stuffed *paratha, idli, dosa, bada, upma, poha, jalebi*, sweets, non-veg items, roasted veg and non-veg items, tea/coffee, *lassi*, fresh fruit juice, lemonade, milk shake, soup	[57]
**Fast foods**	Carbonated drinks, and shakes	Fruit juices (if freshly prepared), milk	[58]
	Pizza, burger, chocolate, ice cream, cookies/cake, *chowmein*, and *Maggi*.	Pasta	[59]
	Chocolates/pastries, pickles, biscuits or other bakery items like bread, toast, buns, cold drinks/soft drinks, potato chips/RTE *namkeens*, ice cream/milkshakes, cheese, pizza/burger/frankie or any other fast food including Chinese food	Sweets, *papads* and pickles (if freshly prepared), *namkeens* (freshly prepared)/deep-fried snacks, *paneer*, butter	[60]
	Pizza, burger, chocolates	*Samosa* (if freshly prepared)	[61]
	Fizzy drinks, mocktails, slushy, milkshakes, pizza, French fries, burger, sub, rolls, cakes/pastries	Tea, coffee	[62]
	Pastries, pizza, French fries, cheese items, Chinese food, soft drinks, sweetened fruit drinks, energy drinks	Coffee/tea	[63]
**Fast foods, junk foods, instant foods, street foods**	**Fast foods**: Burgers, pizzas, fries, hamburgers, patties, and nuggets	*Pakora, samosa, namkeen* etc. (if freshly prepared)	[64]
	**Junk foods**: Chips, chocolate, ice cream, soft drinks, and sandwich	-	
	**Instant foods**: Noodles, flavoured corn flakes, and soup powder	Corn flakes (plain, not flavoured)	
	**Street foods**: *Chaat, gol gappa, samosa, tikki*, noodles, *chowmein*, and *vada pav*	*Chaat, gol gappa, samosa, tikki*, (if freshly prepared)	
**Convenience foods**	RTE *dosa*, RTE *idli*, RTE *chapathi* and RTE *parathas*, RTE *upma*	-	[65]
**Modern foods**	Bread	Fruits and juices (freshly prepared), cereals, eggs, oats	[65]
**Western fast foods**	Pizza, burger, French fries, doughnuts, hot dog, noodles	-	[57]
**Food, snacks and beverage**	Breakfast cereal, bread/toast, biscuits/cookies, cake/pastries, French fries, packaged potato chips, pizza, burger, ice cream, ice candy, chocolates, vegetable roll/wrap, chicken roll/wrap/nuggets, egg roll, popcorn, noodles, health drinks (*Bournvita/Horlicks*), soft drink (Sprite, Coke, Pepsi), energy drink (Red Bull, Gatorade)	Breakfast cereal (plain), tea (black tea/milk tea/Irish tea), coffee (cold coffee/black coffee)	[66]
**Instant food products**	*Rasam* mix, lemon rice mix, *puliyogare*, brinjal *kulambu* mix, pasta, *sambhar* rice and masala, *pulao* mix and *paneer* butter masala	-	[67]
**Soft drinks**	Pepsi, Fanta, Slice, Maaza, Coca-Cola	-	[68]

The online grocery retailer scan, label readings of 375 packaged foods were completed (atleast 3 brands per product) and 81 of those food products qualified as UPFs. Several of the packaged Indian traditional foods and snacks such as bottled and packaged pickles, *namkeens* (cereal and pulse-based extruded snacks), *papads*, frozen non-vegetarian meals and snacks, and frozen RTE meals (like *rajmah curry* and rice, *biryani*,* dal makhni*, etc.) had UPF ingredients and additives in their formulation that qualified them as UPFs (Table 3). Food products such as RTE breakfast cereals (e.g. *poha*,* upma*, etc.), RTE Indian curries (e.g. *paneer makhani*, butter chicken, etc.), Indian RTE bread (e.g. *thepla*, *paratha*, etc.), RTC mixes (e.g. *idli* mix, *dal vada* mix, etc.) also qualified as UPFs. However, some RTE traditional meals such as RTE *biryani*, RTE *rajmah* curry with rice, RTE *kadhi pakoda* with rice were not categorized as UPFs as these did not include UPF ingredients.

**Table 3:** List of traditional Indian snacks/meals and specific ingredients present that qualify them as UPFs

Traditional Indian snacks/meals	Ingredients that qualify for UPFs
Pickles	Preservative (INS 211), Capsicum Extract, Paprika Extract
*Namkeens*	Maltodextrin, Dextrose, Milk Solids, Flavor Enhancers (INS 627 and INS 631), Citric Acid (INS 330), Paprika Extract (INS 160 C), Tomato Powder, Tamarind Powder, Date Powder, Anticaking Agent (INS 551), Spinach Powder, Edible Starch, Potato Solids, Natural [E100 and E141(ii)] and Synthetic food colour (E133 / FD & C Blue No.1) and Added flavour (Nature identical flavouring), Leavening Agent (INS 500 (I), Synthetic Food Colours (INS 133), Compounded Asafoetida [Carrier (INS 414), Soy Lecithin (E322)
Frozen non-vegetarian foods/snacks	Emulsifiers [INS 451 & INS 450], Sugar, Antioxidant [INS 250], Milk Solids, Modified Starch, Milk Protein, Stabiliser (sodium triphosphate), Onion extract, Spice extracts, Preservative (sodium nitrite), Soya Protein, Texturizer (INS 451), Preservative (INS 250), Color Fixative (INS 250), Raising Agent (INS 500), Maltodextrin, Wheat Fibre
*Papads*	Raising agent [INS 500 (ii)], Preservatives (INS 200), Raising agent [INS 500 (i) and INS 500 (ii)], Sodium bicarbonate [E 500 (ii)]

Consumer preferences for the confirmed UPF food categories identified above, were assessed using saliency scores. Table 4 lists these categories and shows the order of preference based on the saliency scores. The last column in the table indicates ‘frequently’, ‘infrequently’ or ‘rarely’ preferred UPFs by consumers in India. The frequently preferred UPFs were breads, chips and other extruded snacks (such as potato chips, cheese balls, puff corns, etc.) and sugar-sweetened beverages (such as cold drinks, diet coke, sodas, and energy drinks. The three rarely preferred UPFs were margarine and frozen/ packaged vegetarian and non-vegetarian snacks and meals (such as stuffed/plain *parantha*,* naan*,* palak paneer*,* rajma*, cutlets, fish/seafood snacks, salami, and sausages).

**Table 4:**
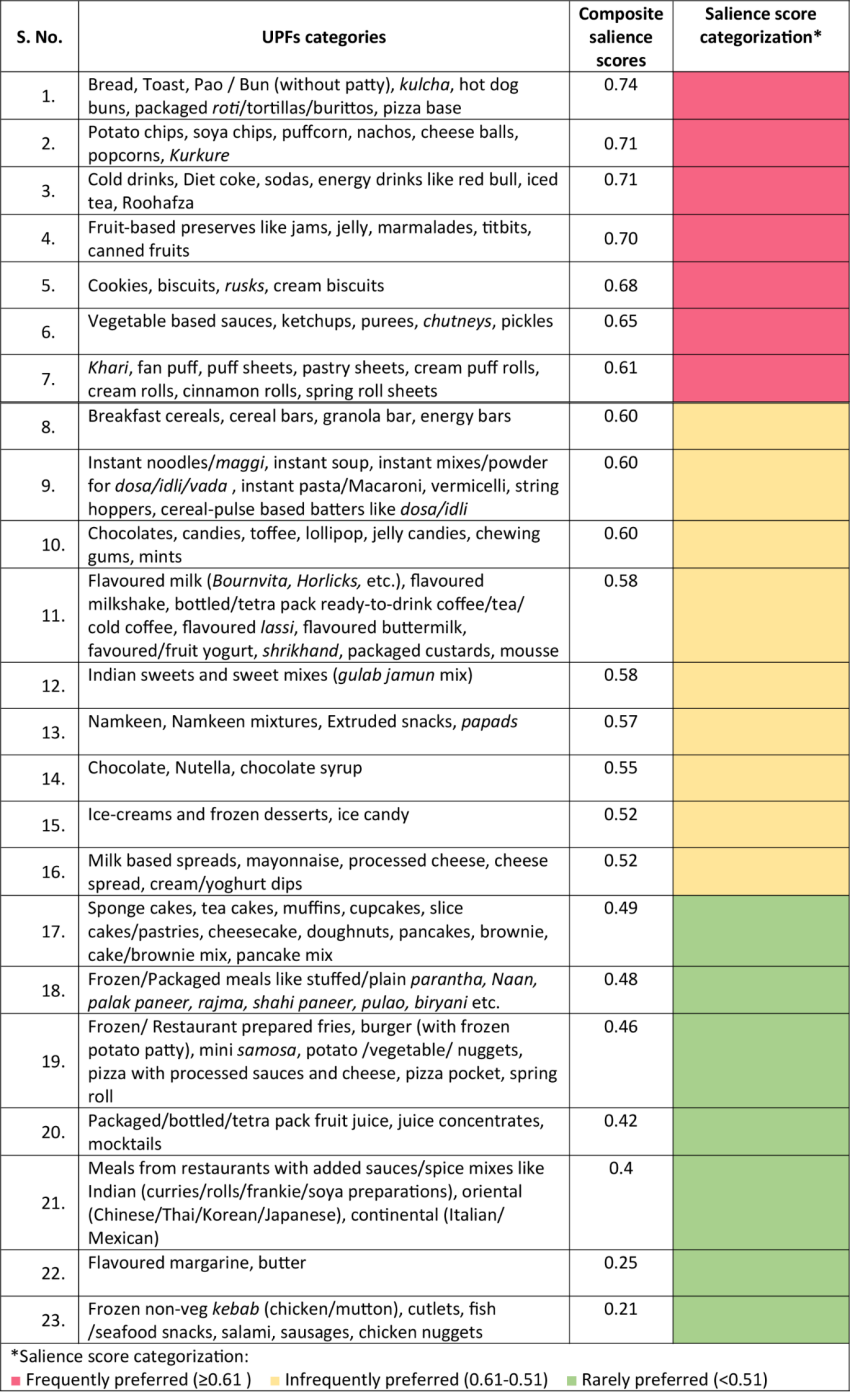
Preference ranking of 23 categories of UPFs among the Indian population, estimated based on saliency scoring (n = 23 research articles)

## Discussion

The present study aimed to identify the specific categories of UPFs accessed in India and rank them by consumer preference using a literature review, an online grocery retailer scan and saliency analysis. We found 23 categories of UPFs accessed by Indian consumers. After analysing the ingredient list of UPFs, we found that product formulation of several traditional Indian foods has transitioned from being processed to ultra-processed category with the use of industrially processed ingredients and presence of additives such as artificial colours, flavour enhancers, anti-caking agents. These ultra-processed versions of traditional foods even though have similar nutrient composition to home-prepared meals, are increasingly consumed, displacing home-cooked meals, and substituting staples. While the health effects of this displacement from minimally processed food ingredients to UPFs is an area of on-going research, we have growing evidence that UPF dietary patterns are linked to poor health outcomes [23, 69]. It is crucial to track reformulation of traditional recipes to ultra-processed convenience foods especially since traditional meals are thought to be healthier [70]. The increasing market of ultra-processed traditional Indian recipes with poor nutritional profile needs more scrutiny and research.

Saliency analysis identified the preferred UPFs among the Indian population with breads, chips and sugar-sweetened beverages being the most preferred UPFs and frozen non-vegetarian snacks being the least preferred. This finding is consistent with the sales trends reported by Euromonitor International in 2020, which has also highlighted a substantial contribution of similar categories of packaged foods, such as bakery items, biscuits, packaged dairy products, savory snacks, and sauces and condiments [46]. Further, saliency analysis also indicates the preference of Indian consumers towards UPFs such as fruit-based preserves, cookies and biscuits, Indian sweet mixes, sauces and pickles, instant noodles/soups/ pasta and savoury puff rolls. Studies from other low and middle-income countries (LMICs) demonstrates similar trends in preference (consumption of UPFs and contribution to percentage of total calories) towards packaged confectioneries, savoury snacks, deep-fried foods, biscuits, candy/ chocolate, savoury snacks, canned red and luncheon meats, pre-fried French fries, mayonnaise, ketchup, fast-food such as sandwiches and pizzas, chips and salty snacks (including tortillas and pretzels), sweets and sweetened beverages and sausages (including canned) [71, 72].

Our results also suggest a benefit of utilizing a classification system based on processing. Currently several UPFs are being captured by proxy descriptors like junk foods, fast foods, convenience foods, instant foods, packaged foods, etc. This limits comparability with other studies, monitoring the preference for and consumption of these products by the population, developing targeted interventions, tracking product reformulation and other regulatory measures to control exposure of these foods to vulnerable age groups through food advertising, etc. [73]. Using UPFs more consistently in studies reporting unhealthy food consumption pattern in India will help with global comparisons and in also elucidating the health effects of these foods. Additionally, as per the packaged food sales data from 2015-19, the Indian UPF market is slowly expanding with increasing sales of RTE meals, savoury snacks, processed fruits, vegetables, meats and other packaged foods [46]. The Nova food classification system can serve this purpose and may be explored as an option for categorization of foods by regulatory authorities. This classification system is used to assess dietary patterns in several high and middle-income countries [23, 70]. Food based dietary guidelines of several countries such as Brazil, Uruguay, Ecuador, Peru and Israel have utilized Nova classification system to inform their dietary recommendations [74–78].

The present paper identified only a limited number of Indian studies which were primarily reported from 2 geographical regions. More such surveys on the consumption of UPFs are desirable to identify common regional UPFs. In the Indian context, several UPFs are indigenously produced by local retailers apart from the huge market share of nationally known branded UPFs [79]. These locally accessible UPFs have greater penetration into the local markets.

The categories of UPFs in India developed in the present study after due validation can be developed into a UPF consumption screener. This tool can be used for monitoring the UPF consumption in India and can address critical gap in scientific literature. This information on quantitative estimate of UPF consumption among Indian population can be useful for assessing impact of UPF consumption on increasing burden of NCDs in India.

### Strengths

This study is one of the first attempts to explore the types of UPFs in the Indian food market, identify the types of packaged traditional recipes that have been converted to UPFs, and map their saliency.

### Study limitations

Studies reviewed were majorly from South India and largely represented the urban population, hence the results cannot be extended to the rural population. The study could only conduct saliency mapping of preferred foods without quantity of intake of UPFs and their contribution to total day’s energy intake. We could not explore traditional variants of UPFs that may be sold in the local unregulated markets.

## Conclusions

India needs to develop a food classification system while systematically defining food categories based on level of processing. This should be followed by an assessment of the extent of UPFs consumption in India. The mapping of the UPFs in India reported in this paper provides the first step in developing a quick screener that systematically lists all the UPF categories. The data generated on consumption of UPFs using the screener is likely to inform policies on regulating the Indian UPFs market, undertake consumer education initiatives and create nutrition literacy around UPFs and thus contain their indiscriminate consumption. This may address the impact of UPF consumption on increasing burden of NCDs in India. There is an urgent need for strengthening the food regulatory environment to check the infiltration of several unhealthy UPFs in the Indian food market.

### Electronic supplementary material

Below is the link to the electronic supplementary material.

Supplementary Material 1

Supplementary Material 2

## Data Availability

No new data was created or analyzed under the literature review part of the study. The datasets used as part of a particular component is available from the corresponding author on reasonable request.

## Abbreviations

UPFs: Ultra-processed foods
NCDs: Non-communicable diseases
DR-NCDs: Diet-related non-communicable diseases
FFQ: Food frequency questionnaires
HFSS: High fat sugar salt
LMICs: Low and middle-income countries
RTE: Ready-to-eat
RTC: Ready-to-cook
